# Functional Implications of Biochemical and Molecular Characteristics of Donation After Circulatory Death Livers

**DOI:** 10.1097/TXD.0000000000000527

**Published:** 2015-06-15

**Authors:** Ryota Masuzaki, Hui Yu, Philip Kingsley, Lawrence Marnett, Zhongming Zhao, Seth J. Karp

**Affiliations:** ^1^ The Transplant Center, Department of Surgery, Vanderbilt University Medical Center, Nashville, TN; ^2^ Department of Biomedical Informatics, Vanderbilt University School of Medicine, Nashville, TN; ^3^ Vanderbilt-Ingram Cancer Center, Vanderbilt University School of Medicine, Nashville, TN; ^4^ Vanderbilt Institute of Chemical Biology, Vanderbilt University School of Medicine, Nashville, TN; ^5^ A. B. Hancock, Jr., Memorial Laboratory for Cancer Research, Vanderbilt University School of Medicine, Nashville, TN; ^6^ Department of Cancer Biology, Vanderbilt University School of Medicine, Nashville, TN

## Abstract

In aggregate, livers donated after circulatory death (DCD) provide lower rates of graft and patient survival compared to brain dead donors (DBD). A method to identify DCD livers likely to perform well would lead to better decision-making regarding which livers to use and which to discard and is an important unmet clinical need. We hypothesized that the ischemic time between extubation and cold perfusion in the donor leads to immediate and unique biochemical and molecular changes that could be used to predict subsequent function.

**Methods:**

Biopsies from normal perfused liver, immediately after cold perfusion during DCD or DBD liver procurement, and during subsequent cold storage were analyzed and compared. Biochemical analysis included adenosine triphosphate (ATP), adenosine diphosphate, adenosine monophosphate, hypoxanthine, xanthine, inosine, nicotinamide adenine dinucleotide, and flavin adenine dinucleotide. Levels of these metabolites were compared to peak posttransplant aspartate aminotransferase as a marker of ischemic injury. Molecular analysis was performed by transcriptional profiling using high throughput sequencing.

**Results:**

Immediately after cold perfusion in the donor, biochemical analysis revealed lower levels of ATP and adenosine diphosphate in DCD versus DBD liver samples (*P* < 0.01 in both cases). The ATP levels showed high negative correlation with peak aspartate aminotransferase levels in recipients (*P* = 0.029). Four hundred seventy genes showed differential expression in DCD but not DBD samples immediately after cold perfusion compared with normal liver samples. Upregulated genes function in inflammation and immunity, whereas downregulated genes function in translation. During cold storage, samples were transcriptionally inactive with no consistent changes in messenger RNA expression.

**Conclusion:**

The ATP content of liver samples taken immediately postperfusion correlates with ischemic injury. Transcriptional profiling identifies biological process that may be relevant for enhancing function in DCD liver transplantation. Transcriptional inactivity of cold stored samples suggests messenger RNA levels over time are unlikely to provide prognostic data.

## Abstract 2

Supplemental digital content is available in the text.

Liver transplantation provides outstanding outcomes and quality of life to patients with end-stage liver disease. Unfortunately, the shortage of organs results in more than 3000 deaths per year in the United States alone (http://optn.transplant.hrsa.gov/latestData/rptData.asp).

One way to reduce wait list deaths is to increase the number of grafts used for transplant. Current methods to predict organ performance are imperfect and rely on donor history, laboratory values, graft appearance, histology, duration of profound hypotension in the donor after withdrawal of support, and duration of cold storage time.^1,2^ A test to more accurately identify livers likely to provide good function, or conversely, those likely to fail would be an important advance. Such a test would be particularly useful for livers from donors after circulatory death (DCD). Recent data demonstrate that DCD is an independent risk factor for poor outcomes after liver transplantation.^3^ The DCD organs have increased rates of primary nonfunction and ischemic cholangiopathy (IC) compared to donation after brain death (DBD) transplantation.^4^ These patients generally require retransplant. With increasing regulatory oversight and anecdotal poor experience with DCD livers, the total number of DCD livers discarded in the United States rose from 22% to 32%, and since 2002, the total number of DCD livers transplanted decreased.^5,6^ On the other hand, a number of centers continue to use DCD livers with excellent results, in many cases with outcomes equal to DBD.^7,8^ This disparity suggests that criteria to predict subsequent function are not optimal. With current decision-making tools, it is likely that incorrect decisions are being made with useable livers being discarded and unusable ones being implanted.

A diagnostic test to predict the degree of injury of DCD livers would allow better prediction of outcomes. We therefore performed a biochemical and molecular analysis of DCD compared to DBD livers to define parameters that might predict a successful transplant. We found that adenosine triphosphate (ATP) levels immediately postperfusion distinguished between DCD and DBD organs and were highly correlated with peak aspartate aminotransferase (AST) levels in the recipient. Transcriptional profiling revealed a DCD signature that may provide insight into processes occurring during the ischemic period.

## MATERIALS AND METHODS

### Sample Collection

Experimental protocols were approved by the Vanderbilt University Medical Center and Tennessee Donor Services Institutional Review Boards. As part of the consent process for organ donation (for example, first person consent via driver's license, or family consent at the time of death), all donors also consented to research use of the organ, and all recipients underwent informed consent. All donors and recipients were included in the study without exclusion. Biopsies were taken according to standard protocols. To establish a normal baseline for reference, a liver biopsy was obtained in 4 DBD donors upon opening the abdomen. Although transcriptional changes are associated with brain death,^9^ only a few genes are affected, and we did not feel this would invalidate our results. Given the small but nonzero risk for bleeding and donor hypotension after a percutaneous liver biopsy, a separate reference pre-perfusion biopsy was not taken in DCD donors. For DBD donors, a sample was taken after 3 liters of aortic and 2 liters of portal flush with University of Wisconsin solution. For DCD donors, the biopsy was taken immediately after 4 liters of aortic flush with University of Wisconsin solution. For the time course, samples were stored on ice for up to 6 hours and an additional biopsy was cut from the original sample at the indicated time points. We halted the analysis at 6 hours based on preliminary data showing no changes after this time. In total, all 10 patients who received transplants using livers analyzed in the study were followed.

### Clinical Definitions

Model for End-Stage Liver Disease (MELD) score was MELD at transplant and included exception points. Cold ischemic time was from cold perfusion in the donor to reperfusion of the portal vein in the recipient. Warm ischemic time began when the liver was taken out of cold storage and ended when the portal vein was reperfused.

### Biochemical Analysis

The ATP, adenosine diphosphate (ADP), adenosine monophosphate (AMP), hypoxanthine, xanthine, inosine, xanthosine, adenine, adenosine, deoxyadenosine, FAD and NAD were obtained from Sigma (St. Louis, MO).

High-pressure liquid chromatography with UV detector analysis was performed on a system consisting of a Waters 1525 Binary Pump in-line with a Waters 717 Autosampler and a Waters 2996 Photodiode Array detector. Data were processed using Waters Empower software (Waters Corp., Milford MA).

The ATP and its metabolites were analyzed from human liver samples that were frozen immediately upon collection. Each sample was removed from −80°C storage, weighed, and immediately homogenized in 6 N ice-cold perchloric acid in a volume 20× the sample mass. The homogenate was neutralized with 1/2 volume of 1 M K_2_HPO_4_ (pH 11.2). The solution was centrifuged at ∼13,000 rpm. The supernatant was filtered through an Ultrafree centrifugal filter (EMD Millipore, Billerica, MA) and frozen at −80°C until analysis via HPLC-UV.

Immediately prior to HPLC-UV analysis, the samples were removed from −80°C and diluted 3:1 in mobile phase component A (0.13 M sodium phosphate (monobasic) and 0.13 M sodium phosphate (dibasic), with the pH 5.0). Analytes were separated by reverse-phase chromatography on a Phenomenex Polar-RP column (25 × 0.46 cm, Phenomenex Corp, Torrance, CA) using a gradient system. Specifically, the column was held at 0% B (100/80/50/30 (v/v) of deionized water/methanol/acetonitrile/component A) for 5 minutes, then %B was increased to 30% over the next 8 minutes, then B was increased again to 100% over the next 7 minutes and held at 100% for 6 minutes. The column was reequilibrated at initial conditions for 9 minutes prior to each injection. The flow rate was 0.8 mL/min.

The Photodiode Array was programmed to collect data from 210 to 375 nm. Chromatograms at 259 nm were prepared for analyte quantitation. Each analyte was quantitated against a standard curve prepared at neutral pH and run concurrently with the study samples. The UV spectra of the selected analytes was examined and compared to standards to confirm identity.

### RNA Collection and Extraction

RNA was isolated using a Qiagen miRNeasy kit. Complementary DNA was synthesized and extracted using a QiaQuick PCR Extraction kit (Santa Clara, CA), ligated with adaptors, and sequenced on Illumina HiSeq 2000 sequencers.

In total, 64 samples were collected. These included baseline samples immediately before aortic cross clamp in the donor and at various time points during cold storage. Samples were sequenced in 4 batches. Quality control metrics used included index read accuracy, Phred score, and total number of reads. This led to 9 samples from batch I being excluded from the data analysis.

The final analysis used 4 normal preperfusion samples before cold perfusion, 6 DCD, and 10 DBD donors at various time points after cold perfusion for a total of 55 samples. As no data were available to predict the magnitude of changes in parameters evaluated to perform a power analysis, these numbers were chosen based on sample availability and cost of running the analyses. At all times, the statistical analysis took into account the actual number of samples used.

### Messenger RNA-Seq Analysis

Single-end 51-bp reads were mapped to the human reference genome (hg19) using TopHat v. 2.0.8.^10^ Mapping parameters allowed at most 2 mismatches and 20 multihits per read. Samtools (v 0.1.19)^11^ and RNA-SeQC (v 1.1.7)^12^ were used for a mapping summary and quality assessment. Gene-wise read counts were quantified using the HTSeq Python toolbox (version 0.5.4).^13^ Before data normalization, an expression-based gene filter was used to select only genes for which the number of samples with nonzero read count exceeded the number of involved subjects. For cold-storage analysis, we converted read counts to a log_2_ scale and performed a batch-effect adjustment using the ComBat method^14^ from the sva R package (v3.6.0, http://www.bioconductor.org/packages/release/bioc/html/sva.html). Batch-effect adjusted data were converted to count per million values. For preperfusion analysis, there were fewer samples in each batch compared to the cold-stored samples. For this reason, we did not apply the batch-effect adjustment and instead normalized the read count values based on the trimmed mean of M values.^15^

Differential expression analyses used the negative binomial generalized linear model^16^ implemented in the edgeR tool (v3.2.4).^17^ To identify differentially expressed genes, we imposed 2 distinct criteria: (1) a statistical requirement and (2) a consistency check. First, nominal *P* values estimated by edgeR were converted to false discovery rate (FDR) values using the Benjamini-Hochberg method,^18^ and an FDR threshold (≤0.001 or 0.25, for perfusion and cold-storage analyses, respectively) was applied. Second, for each gene, we calculated the logarithm of the fold change values (log_2_FC) for each involved subject and identified subjects with log_2_FC larger than at 0.5. A consistency index was derived for each gene as ratio of subjects with higher-than-threshold log_2_FC values. To ensure consistency, we allowed at most zero inconsistent subject in the perfusion analysis, and one in the cold-storage analysis. Genes with an FDR less than the threshold and those that passed the consistency check were considered differentially expressed.

### Gene Ontology Functional Analysis

To examine the function of genes found to be differentially expressed, we performed gene enrichment analysis using a hypergeometric test based on gene ontology (GO)^19^ terms (in the “Biological Process” group) with R package GOstats.^20^ Genes that displayed differential expression were taken as the reference set. For each term, annotated genes from the reference gene set and the differentially expressed gene set were identified. A *P* value indicative of the enrichment was calculated and further adjusted to FDR using the Benjamini-Hochberg method.^18^ Terms with 4 or fewer observed genes were removed and only leaf terms are reported when FDR was less than 0.001.^21^

## RESULTS

### Biochemical Analysis of DBD and DCD Livers After Perfusion and During Storage

**FIGURE 1:**
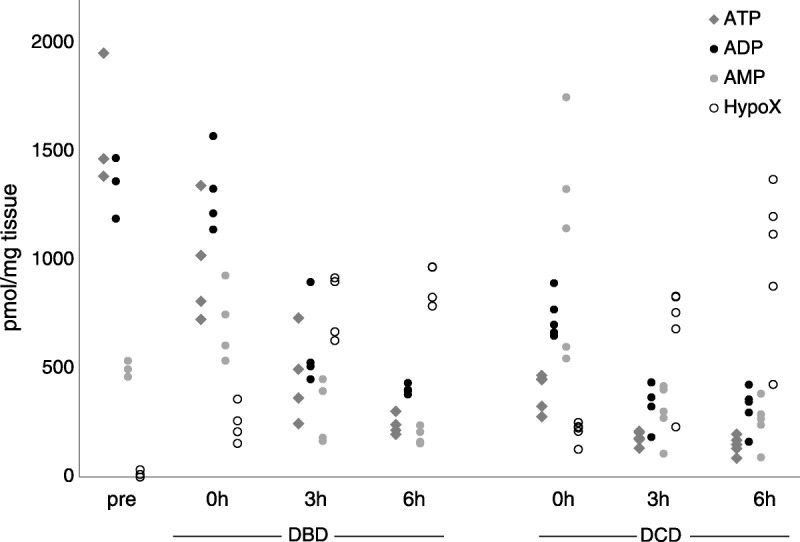
Levels of ATP, ADP, AMP, and hypoxanthine in DCD and DBD livers at baseline and over time. ATP, ADP, and AMP generally decline, whereas hypoxanthine increases after perfusion and during cold storage in both DBD (left middle), and DCD (right) compared to a set of reference values (‘pre’; left side) in normal healthy livers. Immediately after perfusion (0-h), ATP and ADP concentrations were dramatically decreased in DCD compared to DBD livers (*P* = 0.015 and *P* < 0.01, respectively), with no overlap.

To determine the changes in energy substrates over the course of perfusion and cold storage in DBD and DCD livers, we developed an HPLC-based method to examine ATP, ADP, AMP, hypoxanthine, xanthine, inosine, xanthosine, adenine, adenosine, deoxyadenosine, FAD, and NAD. We performed a preliminary analysis with a cohort of 4 DBD and 5 DCD samples to determine changes in these metabolites over time. Figure 1 depicts the changes in ATP (diamonds), ADP (dark circles), AMP (light circles), and hypoxanthine (open circles) over time from preperfusion through 6 hours of cold storage. Compared to a preperfusion baseline (pre, left side) in DBD samples (left middle), ATP levels decreased slightly postperfusion (0 hour, DBD), and continued to fall through cold storage. The ADP levels remained fairly constant between the preperfusion sample (pre) and the postperfusion sample (0 hour, DBD), and declined thereafter. The AMP rose and then declined, whereas hypoxanthine increased between preperfusion and postperfusion, and continued to rise over time in cold storage.

In contrast, in DCD samples (right side), ATP levels dropped dramatically between the baseline preperfusion samples (pre) and postperfusion samples (0 hour, DCD) with no overlap in the samples. The ADP showed a similar pattern, whereas AMP initially increased, and then decayed. Hypoxanthine increased consistently over time after cold perfusion and during storage. Differences in both ATP and ADP levels between DBD and DCD samples were highly significant (*P* = 0.015 and <0.01, respectively). Other metabolites did not show consistent patterns.

**FIGURE 2:**
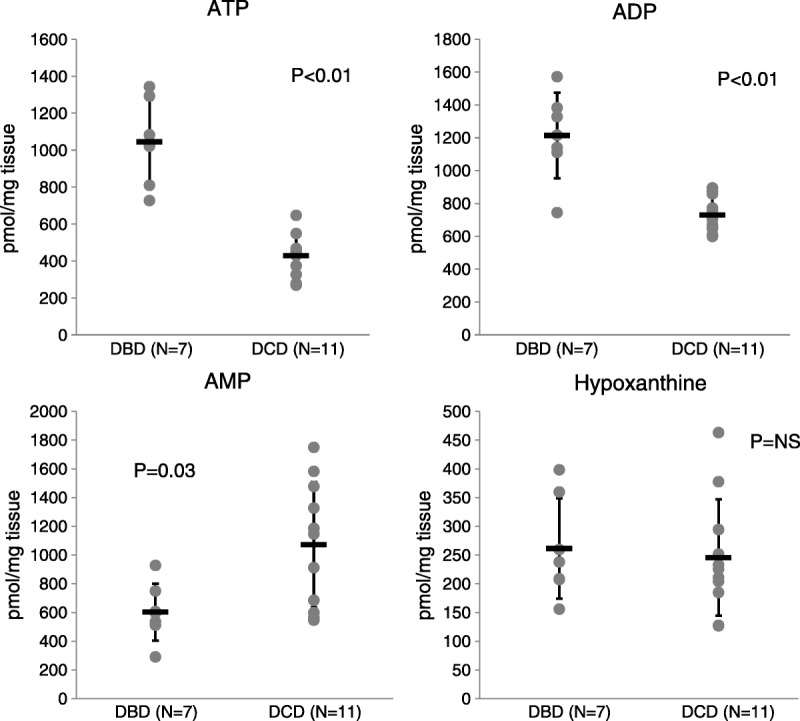
Levels of ATP, ADP, AMP, and hypoxanthine in DCD and DBD livers after cold perfusion in a larger cohort. Immediately after cold perfusion, ATP and ADP are lower in DCD versus DBD livers (*P* < 0.01 in both cases). AMP is higher in DCD versus DBD (*P* = 0.03) and hypoxanthine is unchanged.

These results suggested that the immediate postperfusion sample was the most clinically promising given that (1) the results were most striking between the DBD and DCD samples, and (2) a test of this sample would not require a delay in the decision regarding whether to use the liver. We therefore sought to determine how robust these results were and expanded the analysis to 18 samples, 7 DBD and 11 DCD, focusing only on the immediate postperfusion time point. For this portion of the analysis, we used all DCD donors sequentially recovered over a period of time. Four were ultimately transplanted and seven were not. Figure 2 shows the ATP, ADP, AMP, and hypoxanthine levels in these samples. The ATP and ADP levels were significantly higher in DBD versus DCD samples (*P* < 0.01 for both). Importantly, there was no overlap in ATP levels between the DBD and DCD groups. The AMP levels were significantly lower in DBD samples (*P* = 0.03). Hypoxanthine levels were not different between DBD and DCD organs.

### ATP Levels Correlate With Peak AST Levels

Robustness of ATP levels for differentiating between DBD and DCD procured livers was consistent with our original hypothesis that ATP would be a reliable marker of ischemic injury. To directly address this, we compared ATP levels immediately postperfusion with peak AST levels in recipients after liver transplantation. We examined all of the livers in the previous analysis that underwent transplantation at Vanderbilt and therefore for which posttransplant laboratory values were available, 6 DBD and 4 DCD transplants. Patient demographics are provided in Table 1. There were no differences in donor or recipient age, MELD at transplant, cold ischemia time, or warm ischemia time between the 2 groups. The 4 DCD livers all had short times from extubation to cold perfusion of between 20 and 23 minutes. There were 2 complications in the DBD group, one narrowed bile duct and one wound infection. In the DCD group, 1 patient died of anoxic brain injury on postoperative day 2.

**TABLE 1:**
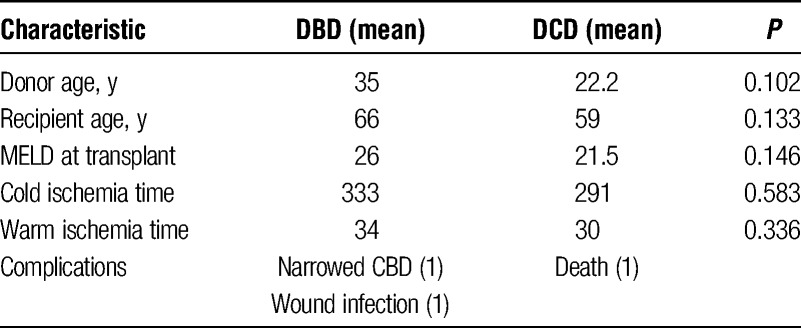
Donor and recipient characteristics

**FIGURE 3:**
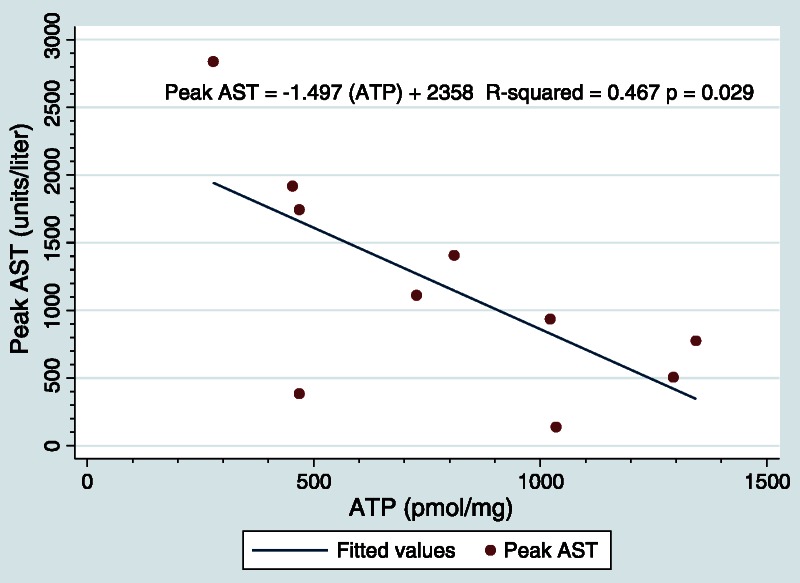
Levels of ATP correlate with recipient AST levels. Plot of ATP levels immediately after cold perfusion in the donor versus peak recipient AST show a strong negative correlation (*P* = 0.0290).

Comparison of ATP levels with peak AST using Pearson statistics revealed a correlation coefficient of −0.683, and R-squared value of 0.467, and a *P* value of 0.029 (Figure 3). This analysis describes that as ATP content of the post-perfusion sample decreases the peak AST increases.

### Transcriptional Analysis of DBD and DCD Livers During and After Cold Perfusion

We hypothesized that the messenger (m)RNA profile of liver samples after cold perfusion could be used to determine important biological processes that occur during DCD procurement and predict subsequent function. To test this, we compared 4 normal, perfused livers against 6 DCD and 4 DBD samples at various times after cold perfusion. On average, 20.0 million raw reads were obtained for each sample. Genome-mapped reads and transcriptome-mapped reads averaged 19.6 and 16.5 million, respectively, corresponding to mapping rates of 98.0% and 84.5%, respectively. Mapping statistics were similar between batches with a minor exception in the 4th batch, which included three of the total four preperfusion samples.

**FIGURE 4:**
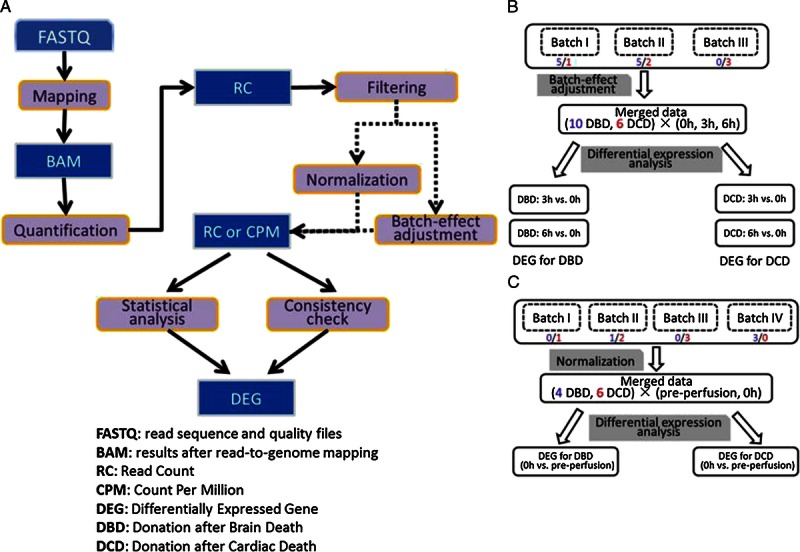
Pipeline for RNA-Seq data processing and differential expression analyses. A, The workflow starts from RNA-Seq raw read files (FASTQ) and ends at expression of differentially expressed genes. Dotted lines indicate different data preprocessing procedures for the perfusion analysis (normalization) and the cold storage analysis (batch-effect adjustment), respectively. B, Sample organization and analysis framework for the cold-storage analysis. C) Sample organization and analysis framework for the perfusion analysis.

Starting from raw read files, read mapping, gene expression quantification, data normalization, and differential expression determination were performed (Figure 4). There were on average 17,122 genes expressed per sample. The adjusted or normalized gene expression data showed a similar distribution pattern across batches (data not shown).

### Differential Expression of Genes Before and After Cold Perfusion

We searched for genes differentially expressed between the normal perfused liver and the immediate DCD or DBD postperfusion sample to look for an ischemic signature present in DCD versus DBD donors. These data might be useful for predicting subsequent function and to describe biological processes that occur during the process of DCD.

**FIGURE 5:**
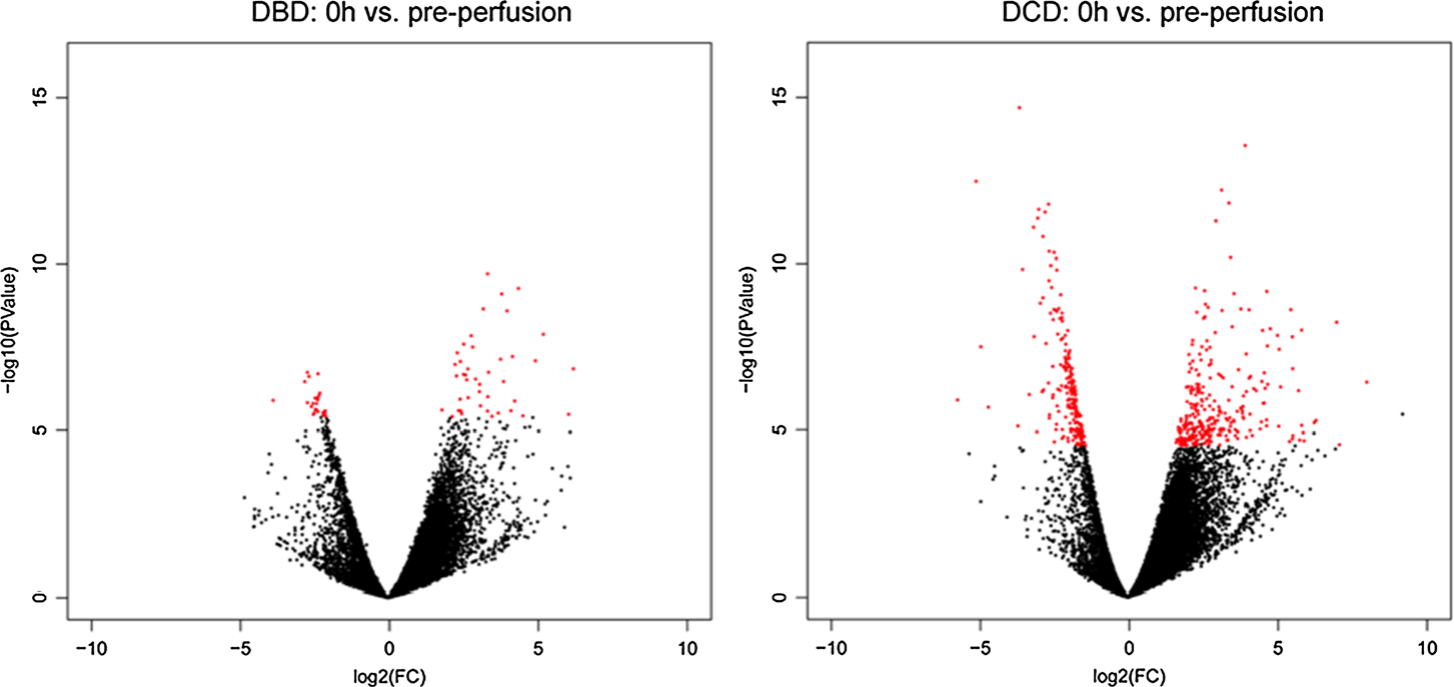
Scatter plot of log fold change versus log *P* value in perfusion differential expression analysis. Under strict conditions, more genes are differentially expressed in DCD versus baseline compared to DBD versus baseline. FC: fold-change. PVal: unadjusted *P* value. Red: differentially expressed genes.

**FIGURE 6:**
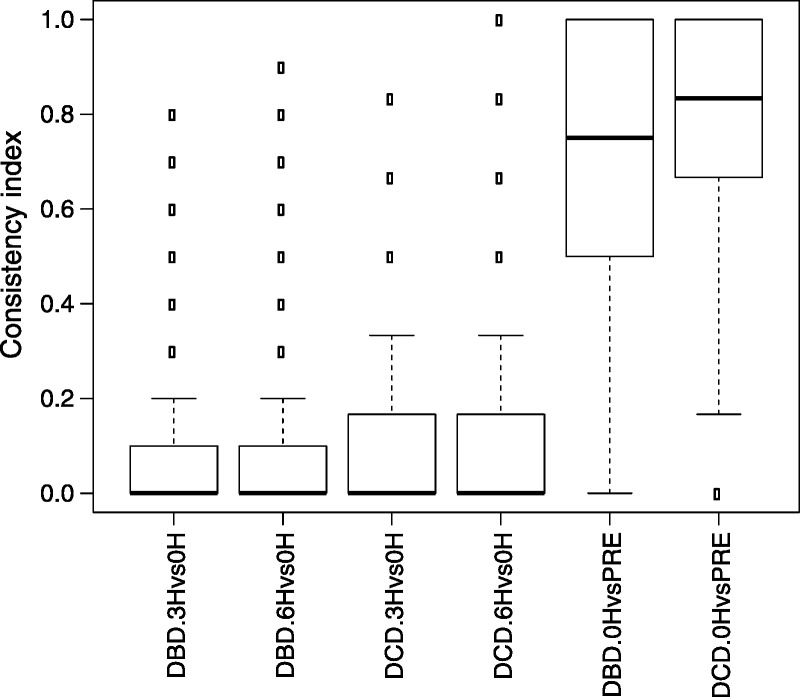
Boxplots of Consistency Index from cold-storage and perfusion comparisons. The applied consistency index thresholds were 0.90 (DBD: 3H vs 0H and DBD: 6H vs. 0H), 0.83 (DCD: 3H vs 0H and DCD: 6H vs. 0H), and 1.0 (DBD: 0H vs. PRE, DCD: 0H vs. PRE). No genes showed consistent change over cold storage.

Because only DBD preperfusion samples could be obtained, they were used as baseline for both DBD and DCD analyses, and a nonpaired comparison test was used for both analyses. A plot of differential expression *P* values versus the mean log fold changes is depicted in Figure 5. We then calculated the consistency indices for each gene, which reflected how the surveyed samples were consistent in showing moderate or higher expression changes of the same direction (up or down). The consistency index distributions were plotted as boxplots in Figure 6.

To be identified as differentially expressed, a gene needed to have an FDR value less than 0.001 and a consistency index of exactly 0, demonstrating high consistency across all samples. To be identified as differentially expressed between DBD and DCD, a gene needed to be differentially expressed in 0-hour DCD samples compared to normal perfused liver, and not differentially expressed in 0-hour DBD livers compared to normal perfused liver.

As expected, we found significant differences in global gene expression after cold perfusion in both DBD and DCD organs (Figure 5, red dots represent differentially expressed genes). Using a stringent and clinically relevant threshold of FDR 0.001 and a subject inconsistency tolerance of 0, we obtained 68 and 514 differentially expressed genes for DBD and DCD, respectively (Table 2). Of these, 470 genes were differentially expressed in DCD but not in DBD samples, 281 upregulated and 189 downregulated (**Figure S1, SDC**, http://links.lww.com/TXD/A3).

**TABLE 2:**
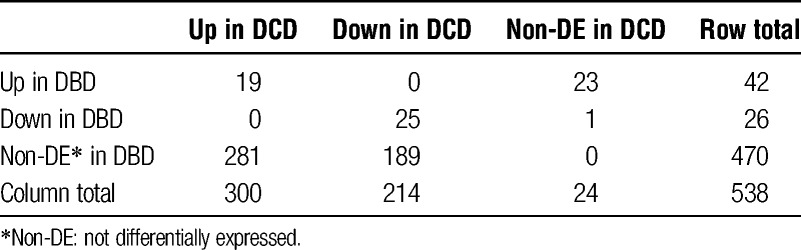
Differentially expressed genes in DBD and DCD samples compared to preperfusion

Genes specifically and differentially expressed in DCD samples were annotated using GO biological processes (Table 3). Many of the significant terms enriched in the upregulated genes were related to inflammation, immunity, and cellular response to damage. Downregulated genes were mostly involved in mRNA translation into protein, with many encoding ribosome proteins.

**TABLE 3:**
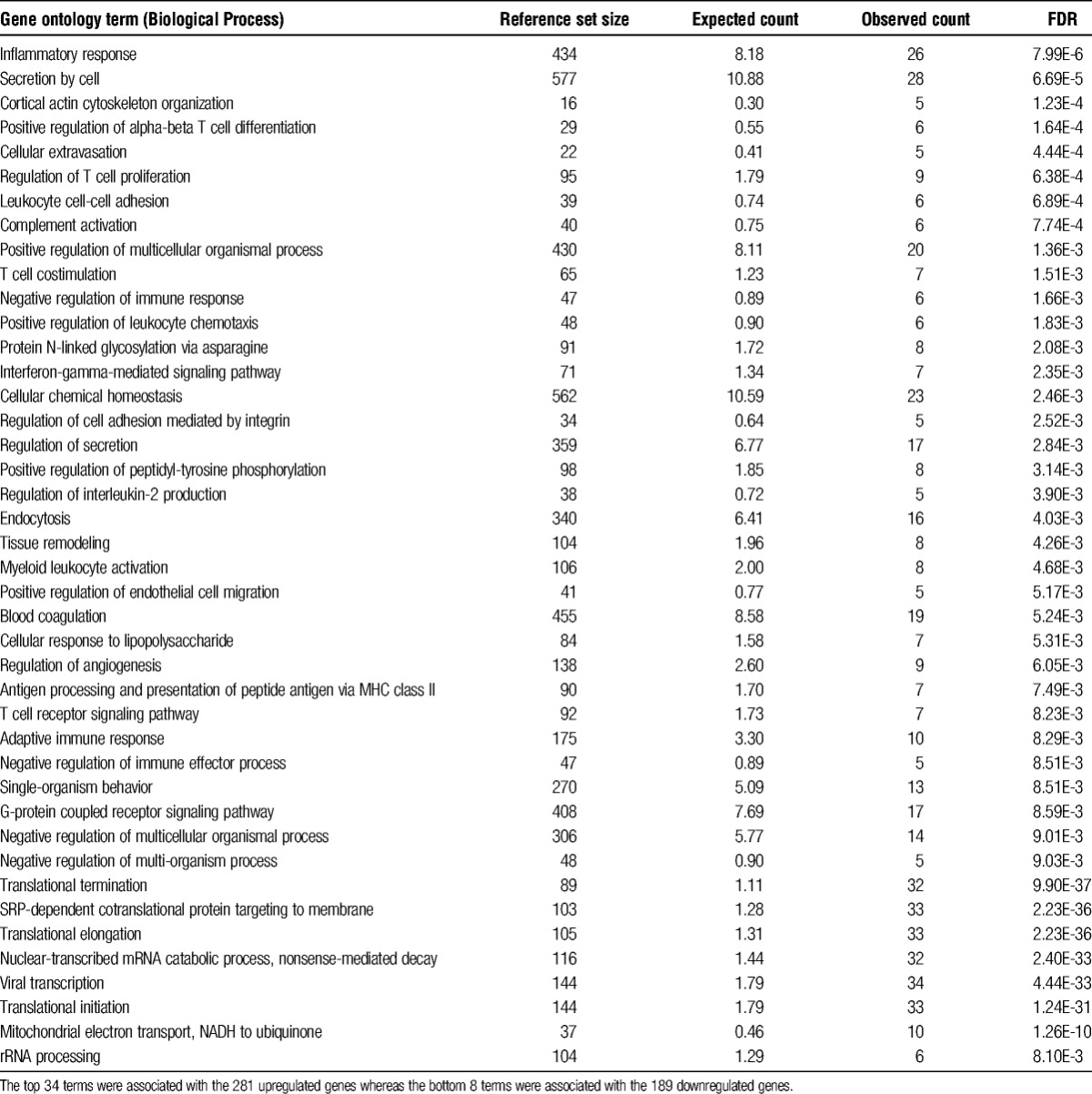
Functional terms enriched within DCD-specific perfusion-responsive differential expression genes

### Cold Storage DBD and DCD Samples Are Minimally Transcriptionally Active

Finally, we examined whether we could find a decay signature over time during cold storage that might help determine the ischemic injury suffered by the liver and/or predict subsequent function. In 6 DCD and 10 DBD samples, tissue was taken immediately after cold perfusion (denoted 0-h) in the donor and then at 3 hours (3-h) and 6 hours (6-h) after perfusion. Each of the 16 donors therefore had 3 independent samples analyzed, for a total of 48 samples.

We compared the expression levels of each gene between the baseline time point (0-h) and a cold storage time point (3-h or 6-h) in the DCD and DBD samples. A donor-paired design strategy was used in which the 0-, 3-, and 6-h time points from the same donor were compared. In other words, for the post-perfusion 3-h time point, we identified 6 pairs of DCD samples and 10 pairs of DBD samples, where each pair of samples came from 1 identical donor but corresponded to 2 different time points (0-h and 3-h). In the differential expression analysis for the perfusion process, we did not pair the samples because in the DCD group, the samples were not paired (4 preperfusion DBD samples vs six 0-h DCD samples). In general, a subject-paired design enables greater statistical power and is advised whenever feasible. At a fairly permissive criterion of an FDR of 0.25 or less, very few genes were found upregulated or downregulated (Table 4). We then considered the consistency index of each gene (Figure 6) and required that the gene be regulated toward the same direction (up or down) in at least 5 of the 6 DCD and 9 of the 10 DBD samples. We added this as recognition that the clinical usefulness of a test like this will depend on high positive and negative predictive values. Using criteria of: 1) a FDR threshold of 0.25 and 2) a subject inconsistency tolerance of 1, not a single gene was found that was differentially expressed in any comparison (Table 4). These results support the conclusion that the cold-stored liver is not transcriptionally active in a consistent and clinically meaningful way.

**TABLE 4:**
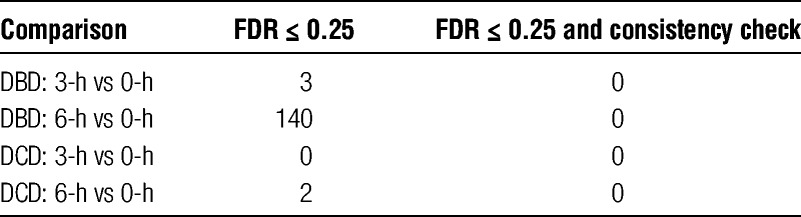
Differentially expressed genes in cold-storage samples

## DISCUSSION

To find parameters that could be used to predict subsequent function of livers procured for transplantation, we describe their biochemical and molecular features at the time of cold perfusion in the donor and during cold storage. Immediately after cold perfusion, ATP and ADP content of the liver reliably distinguishes between DCD and DBD livers, and ATP levels highly correlate with peak AST in the recipient as a measure of ischemic injury. Transcriptional analysis reveals significant upregulation of genes involved in inflammation and the immune response, and downregulation of genes involved in translation. Cold-stored livers are transcriptionally inactive, suggesting that mRNA studies of stored tissue over time are unlikely to provide clinically actionable information.

The most clinically relevant findings are that both ATP and ADP concentrations at the time of cold perfusion are lower in DCD versus DBD livers with no overlap in the samples tested. These results suggest that ATP may be a highly sensitive marker for the degree of ischemia.

This is an important result for 2 reasons. First, it is highly likely that IC, a devastating complication that is more common in DCD versus DBD livers, is caused by ischemia that occurs at the time of procurement. Second, a clinical point of care test to measure ATP and predict liver function could be performed at the time of procurement and not delay use of the organ. This is particularly important for DCD liver transplantation given the importance of minimizing cold storage time. Currently, it is not possible to determine the extent of ischemic injury suffered by the graft, although there are measurements that can be helpful in this regard.^1,2^ A diagnostic test performed immediately after cold perfusion could integrate the damage suffered during the entire process from extubation to cold perfusion. Importantly, the test would have to be able to be performed quickly to provide decision support and not increase cold ischemic time.

Although graft outcomes are clearly dependent on multiple factors related to both the donor and recipient, the goal would be to determine a threshold of ATP above which the liver would be likely to work. This result could then be combined with clinical data for a final determination of whether to use the liver and who would be an appropriate recipient. This information could help counsel the recipient regarding the risk of this particular organ. A reliable test to predict function would decrease rate of primary nonfunction and IC and increase the donor pool by giving clinicians more confidence that outcomes with these livers are predictable.

Changes in ATP and its metabolites are consistent both with what is known regarding energy metabolism and published studies in liver transplantation. During energy production, phosphates on ATP are sequentially hydrolyzed, creating ADP, then AMP, and finally producing hypoxanthine, a breakdown product of ATP. Our results showing decreasing ATP and increasing breakdown products over time after procurement are therefore consistent with ischemic stress after extubation and during storage. These results are consistent with early work by Belzer regarding the importance of maintaining ATP concentrations in tissue.^22^ Our results compliment the finding that increased lactate and lactate/pyruvate ratios in DCD grafts indicate ischemic damage^23^ and that ATP concentrations measure organ viability in DCD rat and pig models.^24–26^

Transcriptional analysis shows the process of death during DCD recovery results in changes in mRNA in the liver. In particular, based on the GO data, inflammation and immune pathways are highly upregulated during the DCD donation process, whereas genes driving general protein production seem to decrease. This injury pattern is not surprising. Others have found microRNA levels change in perfusate and correlate with IC after DCD donation.^27^ These findings may also be of use in predicting primary nonfunction or IC.

It is perhaps surprising that our extensive transcriptional analysis did not produce a reproducible decay signature during cold storage, especially in light of a report to the contrary.^22^ We purposefully chose a high standard for this analysis, and this may explain the discrepancy. This is due to an understanding that the sensitivity and specificity of a test, and not necessarily the *P* value, dictate the clinical utility. The literature is full of tests with very low *P* values that are not used or useful clinically. With the strict criteria we used, not a single transcript was identified. Our analysis does not definitively establish there is no transcriptional activity, just that to our level of analysis the activity is not reliable across all samples. We therefore conclude that following transcription over time during cold storage is unlikely to produce a clinically useful test.

Based on these studies, we feel continued examination in a focused manner examining ATP and ADP concentration and transcriptional changes immediately after cold perfusion in DCD livers is a promising opportunity to evaluate the status of the graft prior to transplant and increase successful use of DCD livers.
